# Environmentally friendly, highly efficient, and large stokes shift-emitting ZnSe:Mn^2+^/ZnS core/shell quantum dots for luminescent solar concentrators

**DOI:** 10.1038/s41598-022-21090-x

**Published:** 2022-10-20

**Authors:** Nyamsuren Byambasuren, A-Ra Hong, Woo-Young Lee, Ji Young Byun, Gumin Kang, Hyungduk Ko, Ho Seong Jang

**Affiliations:** 1 Materials Architecturing Research Center, Korea Institute of Science and Technology, 5, Hwarang-ro 14-gil, Seongbuk-gu, Seoul, 02792 Republic of Korea; 2 Division of Nano & Information Technology, KIST School, Korea University of Science and Technology (UST), Seoul, 02792 Republic of Korea; 3 Nanophotonics Research Center, Korea Institute of Science and Technology, 5, Hwarang-ro 14-gil, Seongbuk-gu, Seoul, 02792 Republic of Korea; 4 Extreme Materials Research Center, Korea Institute of Science and Technology, 5, Hwarang-ro 14-gil, Seongbuk-gu, Seoul, 02792 Republic of Korea

**Keywords:** Quantum dots, Nanoparticles, Solar cells

## Abstract

In this study, heavy-metal-free orange light-emitting ZnSe:Mn^2+^/ZnS doped-core/shell (d-C/S) quantum dots (QDs) were synthesized using a nucleation doping strategy. To synthesize high quality d-C/S QDs with high photoluminescence (PL) quantum yield (QY), the Mn^2+^ concentration was optimized. The resulting ZnSe:Mn^2+^(5%)/ZnS d-C/S QDs showed a high PL QY of 83.3%. The optical properties of the synthesized QDs were characterized by absorption and PL spectroscopy. Their structural and compositional properties were studied by X-ray diffraction, transmission electron microscopy, and energy dispersive X-ray spectroscopy. After doping Mn^2+^ into a ZnSe core, the ZnSe:Mn^2+^/ZnS d-C/S QDs showed a large Stokes shift of 170 nm. The ZnSe:Mn^2+^/ZnS d-C/S QDs were embedded in a poly(lauryl methacrylate) (PLMA) polymer matrix and the ZnSe:Mn^2+^/ZnS-based polymer film was fabricated. The fabricated ZnSe:Mn^2+^/ZnS-PLMA film was highly transparent in the visible spectral region (transmittance > 83.8% for λ ≥ 450 nm) and it exhibited bright orange light under air mass (AM) 1.5G illumination using a solar simulator. The optical path-dependent PL measurement of the ZnSe:Mn^2+^/ZnS-PLMA film showed no PL band shift and minimal PL decrease under variation of excitation position. These results indicate that the highly efficient and large Stokes shift-emitting ZnSe:Mn^2+^/ZnS QDs are promising for application to luminescent solar concentrators.

## Introduction

Luminescent solar concentrators (LSCs) were first reported more than four decades ago and in their basic design, sunlight penetrates the top surface of a polymeric or glass waveguide containing luminescent materials such as organic dyes, inorganic phosphors, and quantum dots (QDs)^1^. The sunlight is absorbed by the luminescent materials embedded in the polymeric or glass waveguide and a fraction of the light emitted from the luminescent materials is guided by total internal reflection to photovoltaic (PV) cells, such as silicon solar cells, which are attached to the edge of the LSCs^1^. The luminescent materials (luminophores) in the LSCs are the most important component of the LSC devices^1^. Among various luminophores, organic dyes such as rhodamines, coumarins, and perylene derivatives, etc., have been widely used in LSCs^2,3^. However, they have a limited absorption bandwidth, a large reabsorption problem due to their small Stokes shift, and stability issue^1^.

Recently, QDs, which are inorganic semiconducting nanocrystals, have been used as luminophores in LSCs because of their high quantum efficiency and higher photostability than organic dyes^4,5^. They also have wider spectral absorption than organic dyes, and absorption and emission bands that are tunable based on the quantum confinement effect^6^. Bomm et al. reported CdSe QD-based LSCs, and Resei’s group reported CsPb(Br_x_I_1-x_)_3_ perovskite QD-based LSCs^6,7^. However, they still exhibit a small Stokes shift, attributed to the large overlap between the excitonic absorption band and the emission band^6,7^.

Several strategies have been introduced to overcome the reabsorption problem resulting from the small Stokes shift of conventional colloidal QDs. These include CdSe/CdS core/thick-shell-structured QDs, CdSe/CdS dot-in-rod structured QDs, type-I CdSe/Cd_1-x_Zn_x_S core/thick-shell-structured QDs, ternary I-III-VI_2_ group QDs (CuInSe_x_S_2-x_ and CuInS_2_) showing defect level-involved emission, and Si QDs with reduced self-absorption due to indirect-band gap of the silicon^8–14^. However, the QDs in most previously reported strategies contain a toxic heavy metal such as cadmium^8–11^. In the case of I-III-VI QDs and Si QDs, there is still an overlap between the absorption and emission bands, although they are environmentally benign and showed an enlarged Stokes shift^12–14^.

In addition to engineering the nanostructures of the QDs and utilizing defect-related emissions from the QDs, doping transition metals or lanthanide ions into the QDs can be another efficient way to enlarge the Stokes shift since doping impurities into the QDs can dramatically change their emission properties^15–18^. Recently, Klimov’ group reported Cd_x_Zn_1-x_S:Mn^2+^/ZnS QD-embedded LSCs and Gamelin’s group reported Cu-doped CdSe QD-embedded LSCs^19,20^. Additionally, Wu’s group reported LSCs using Yb^3+^-doped CsPbCl_3_ perovskite QDs^21^. Although the CdS:Mn^2+^, CdSe:Cu and CsPbCl_3_:Yb exhibited significantly large Stokes shifts, the QDs utilized in the LSCs still contained toxic heavy metals such as cadmium and lead. On the other hand, Patrick’s group applied Cd- and Pb-free Mn^2+^-doped ZnSe QDs to LSCs and reported the optical properties of the fabricated LSC film^22^. While undoped ZnSe-based QDs exhibited a narrow emission band in the deep blue spectral region, ZnSe:Mn^2+^-based QDs showed a redshifted emission band in the orange spectral region, resulting in a large Stokes shift^23^. Even though the previously reported ZnSe:Mn^2+^/ZnS QDs in the fabricated LSC film exhibited nearly zero reabsorption properties due to their large Stokes shift, the ZnSe:Mn/ZnS QDs incorporated into the LSC film had moderate photoluminescence (PL) quantum yield (QY) of ~ 50%^22^. This suggests that enhancing the PL QY of the ZnSe:Mn-based QDs is required for QD-based LSCs.

In this study, we report environmentally benign and highly efficient ZnSe:Mn^2+^/ZnS doped-core/shell (d-C/S) QDs with large Stokes shift. The QDs were synthesized using a nucleation doping strategy with metal oleate precursors such as zinc oleate and manganese oleate. The as-synthesized ZnSe:Mn^2+^/ZnS d-C/S QDs showed bright orange light under ultraviolet (UV) light excitation (PL QY = 83.3%). Importantly, our QDs showed a large Stokes shift where the absorption and emission bands did not overlap at all, unlike conventional QDs such as CdSe^6^. Such a large Stokes shift of the ZnSe:Mn^2+^-based QDs ensures zero reabsorption of the QDs’ emission. We incorporated the ZnSe:Mn^2+^/ZnS d-C/S QDs into poly(lauryl methacrylate) (PLMA) polymer to fabricate a transparent LSC film, and then investigated the optical properties of the ZnSe:Mn^2+^/ZnS-PLMA LSC film.

## Results and discussion

**Figure 1:**
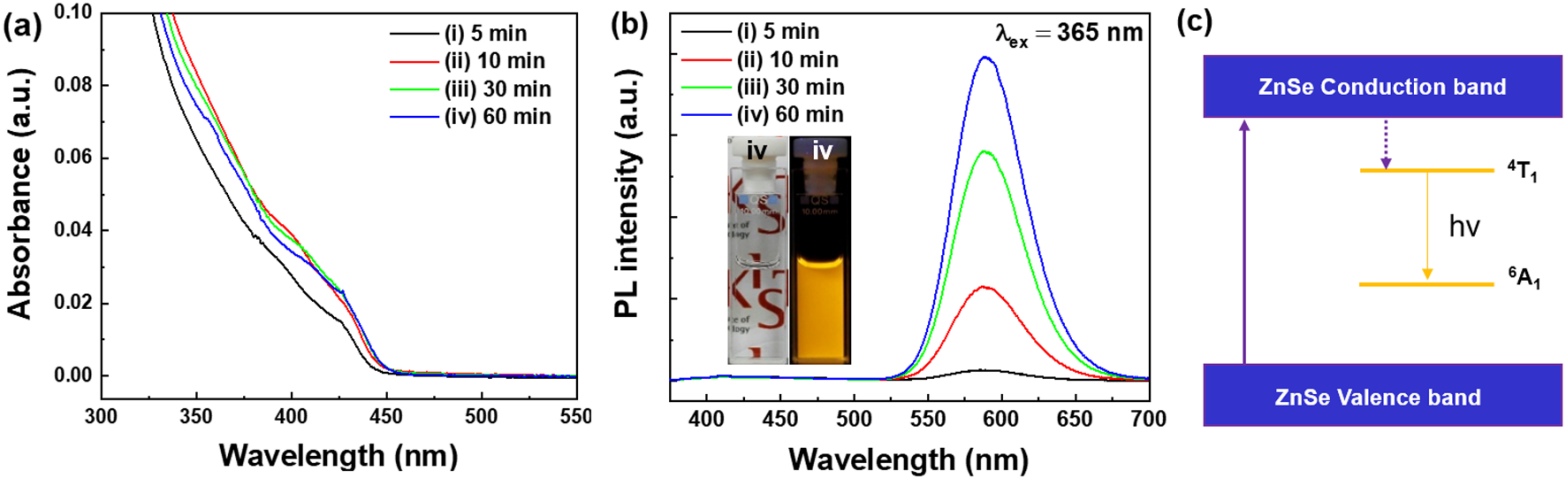
(**a**) Absorption and (**b**) PL spectra of the as-synthesized ZnSe:Mn^2+^(5%) d-C QDs as a function of reaction time (i: 5 min, ii: 10 min, iii: 30 min and iv: 60 min). The inset in (**b**) shows photographs of the ZnSe:Mn^2+^(5%) d-C QDs solution under indoor light (left) and UV light (right) (λ_ex_ = 365 nm). (**c**) Schematic energy level diagram showing the emission from the ZnSe:Mn^2+^ d-C QDs.

Figure 1a shows the absorption spectra of the 0.05 mmol Mn^2+^-doped ZnSe (ZnSe:Mn^2+^(5%)) doped-core (d-C) QDs as a function of reaction time. When the reaction time was 5 min, the absorption spectrum of the ZnSe:Mn^2+^(5%) d-C QDs showed a first exciton peak at 426 nm, which is attributed to the transition of electrons from the valence band to the conduction band^24^. A redshift of the absorption spectra of the ZnSe:Mn^2+^(5%) d-C QDs was observed as the QD particles grew with increasing reaction time. This size-dependent redshift of the absorption band is attributed to the quantum confinement effect^23^. Based on the absorption spectra, it is believed that the growth rate of the ZnSe:Mn^2+^(5%) d-C QDs became slower after 10 min reaction. While the absorption band of the ZnSe:Mn^2+^(5%) d-C QDs showed a redshift from 426 to 430 nm, their PL bands were observed at around 588 nm which is attributed to the d-d transition (^4^T_1_ → ^6^A_1_ transition) of the Mn^2+^ ions^25^. Except for the broad PL band attributed to the d-d transition of the Mn^2+^ ions, no band-to-band emission peak, which could be attributed to the radiative recombination of electrons in the conduction band and holes in the valence band, was observed (Fig. 1b).

When the ZnSe:Mn^2+^ was irradiated with UV light, excited energy was transferred from the host to the Mn^2+^ dopant ions and then the Mn^2+^ ions exhibited broad emissions due to the ^4^T_1_ → ^6^A_1_ transition, as shown in Fig. 1c^26,27^. Due to the broad PL band peaking at 588 nm of the ZnSe:Mn^2+^ d-C QDs, orange emission was observed under illumination with a UV lamp (λ_ex_ = 365 nm), as shown in the Fig. 1b inset. However, the brightness of the orange emission from the d-C QDs was not high, and the absolute PL QY of the ZnSe:Mn^2+^(5%) d-C QDs was measured to be 30.5%.

To enhance the PL intensity, a ZnS shell was overcoated on the ZnSe:Mn^2+^(5%) d-C QDs. It is well-known that forming a ZnS shell on the core can effectively reduce surface defects, resulting in PL enhancement^28^. In addition, the emission from Mn^2+^ dopants is considered to be a result of the energy transfer from the photoexcited host to the Mn^2+^ ions in Mn^2+^-doped QDs^29,30^. Therefore, the formation of the ZnS shell, with a wider band gap than the ZnSe core, leads to enhanced exciton confinement which contributes to efficient energy transfer from the exciton in the host to the Mn^2+^ ions, and finally, the efficiency of the Mn^2+^ emission can be increased^30^. Thus, one can expect that forming a ZnSe:Mn^2+^/ZnS d-C/S structure will result in PL enhancement of ZnSe:Mn^2+^. As shown in Fig. S1, the PL intensity of the ZnSe:Mn^2+^(5%) QDs was significantly increased. It is noted that the peak wavelength of the emission band shifted from 588 nm for the ZnSe:Mn^2+^(5%) d-C QDs to 596 nm for the ZnSe:Mn^2+^(5%)/ZnS d-C/S QDs. This redshift of the PL band is consistent with previous reports^31–34^ and attributed to smaller crystal field splitting of the d orbital of the Mn^2+^ ions and lattice compression caused by the ZnS shells^25,33^.

**Figure 2:**
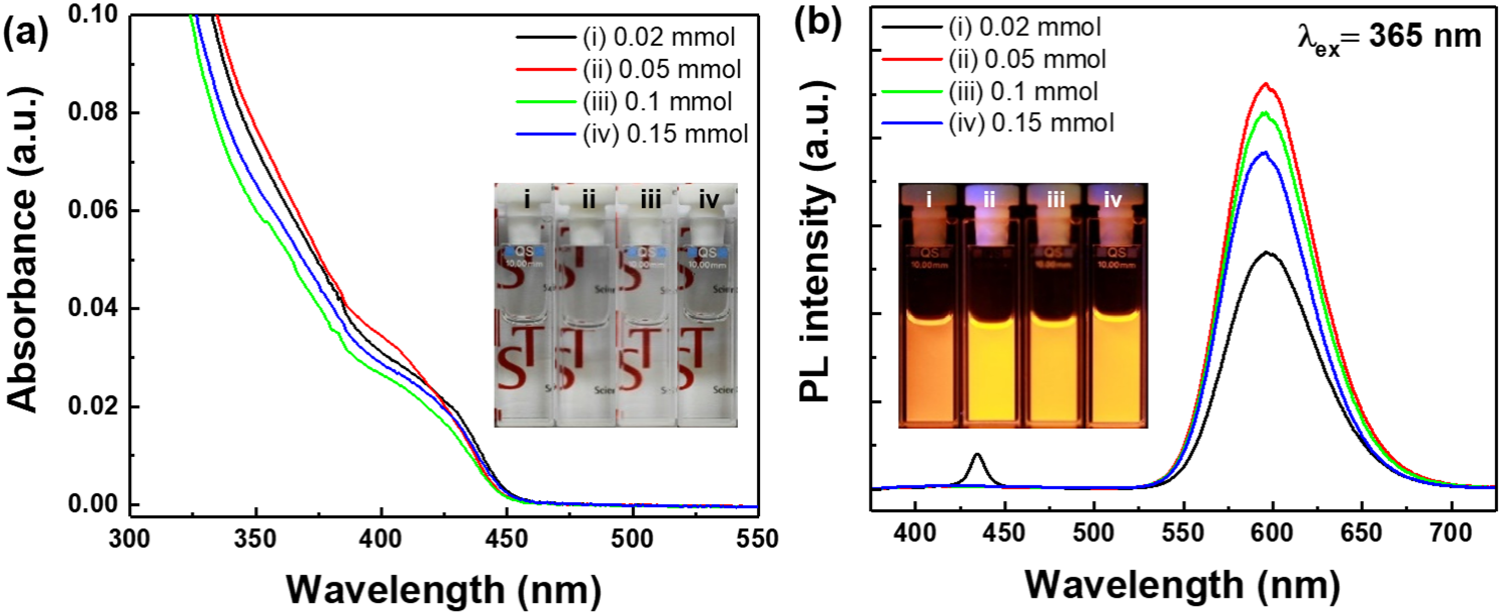
(**a**) Absorption and (**b**) PL spectra of the as-synthesized ZnSe:Mn^2+^/ZnS d-C/S QDs synthesized with various amounts of Mn-oleate (i: 0.02 mmol, ii: 0.05 mmol, iii: 0.1 mmol, and iv: 0.15 mmol). Insets in (**a**) and (**b**) show photographs of the ZnSe:Mn^2+^/ZnS d-C/S QD solutions under indoor light and UV light (λ_ex_ = 365 nm), respectively.

To optimize the Mn doping concentration, the amount of Mn precursor was varied from 0.02 to 1.5 mmol. As shown in Fig. 2a, all of the ZnSe:Mn^2+^/ZnS d-C/S QD samples showed the almost same first exciton absorption peak, indicating the dopant amount did not affect the band gap energy of ZnSe^35^. When 0.02 mmol of Mn(OL)_2_ precursor was added to the ZnSe core, a weak PL band was observed at about 435 nm in addition to the PL band peaking at 596 nm (Fig. 2b). The observed emission bands were attributed to both ZnSe host-related band-to-band emission and the d-d transition of the Mn^2+^ dopant ions^27^. This result indicates that the ZnSe QDs were separately formed besides the ZnSe:Mn^2+^ d-C QDs when the amount of Mn(OL)_2_ precursor was insufficient in our synthetic condition. However, when the amount of Mn(OL)_2_ precursors was larger than 0.02 mmol, only a single emission band from the Mn^2+^ ions was observed at 596 nm. In this study, the optimized Mn(OL)_2_ amount was 0.05 mmol, and the ZnSe:Mn^2+^(5%)/ZnS d-C/S QDs showed the highest PL intensity and the brightest orange light under 365 nm UV light, as shown in Fig. 2b. When we investigated the PL QY of the ZnSe:Mn^2+^(5%)/ZnS d-C/S QDs using an integrating hemisphere, the absolute PL QY of the ZnSe:Mn^2+^(5%)/ZnS d-C/S QDs was measured to be 83.3%. In addition to the high PL QY of the ZnSe:Mn^2+^(5%)/ZnS d-C/S QDs, it is worth noting that the Stokes shift of the ZnSe:Mn^2+^(5%)/ZnS d-C/S QDs was as large as 170 nm, which is much larger than conventional QDs such as CdSe^6^.

**Figure 3:**
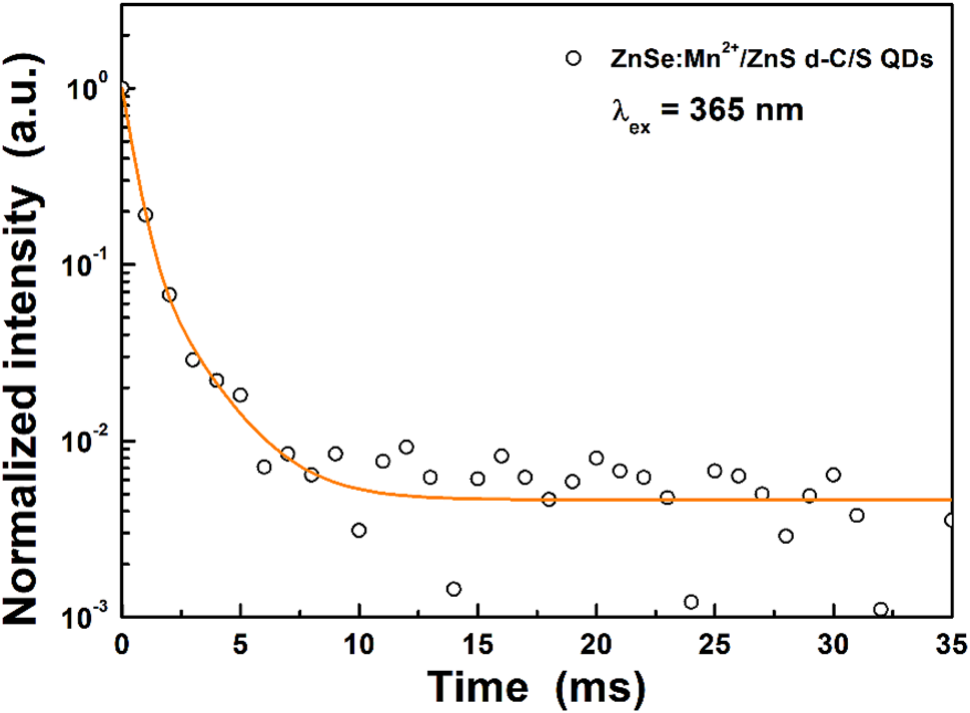
A decay profile of the ZnSe:Mn^2+^(5%)/ZnS d-C/S QDs under 365 nm UV light.

Figure 3 shows the decay profile of the ZnSe:Mn^2+^(5%)/ZnS d-C/S QDs. The luminescence decay from the ^4^T_1_ excited state of the Mn^2+^ ions was monitored for emission at 592 nm under excitation with 365 nm UV light. As shown in Fig. 3, the decay curve was fitted with a biexponential function^36^, and the average decay time (τ_avg_) was calculated with the equation below^32,37^

1 $${\tau }_{avg}=\frac{({A}_{1}{\tau }_{1}^{2}+{A}_{2}{\tau }_{2}^{2})}{({A}_{1}{\tau }_{1}+{A}_{2}{\tau }_{2})}$$

where τ_1_ and τ_2_ are the decay time components, respectively, and A_1_ and A_2_ are the amplitudes of the decay components, respectively. The fast and slow decay components were 0.481 and 1.899 ms, respectively, and the average decay time was calculated to be 1.019 ms. This slow decay time is comparable to those in previous reports^32,38^, and is due to the spin-forbidden ^4^T_1_ → ^6^A_1_ transition of the Mn^2+^ ions^39^. The fast decay component might be related to the exchange coupled Mn^2+^ ion pairs since the exciton recombination and trap emissions show very short decay times, in the range of 10^–9^ and 10^–10^ s, respectively, and the decay time of the Mn^2+^ pairs is known to be shorter than that of a single Mn^2+^ ion^36,40,41^. The slow decay component is attributed to the emission from the isolated Mn^2+^ ions in the ZnSe lattice^36^. The orange emission and long decay time support successful Mn^2+^ doping into the ZnSe core QDs.

**Figure 4:**
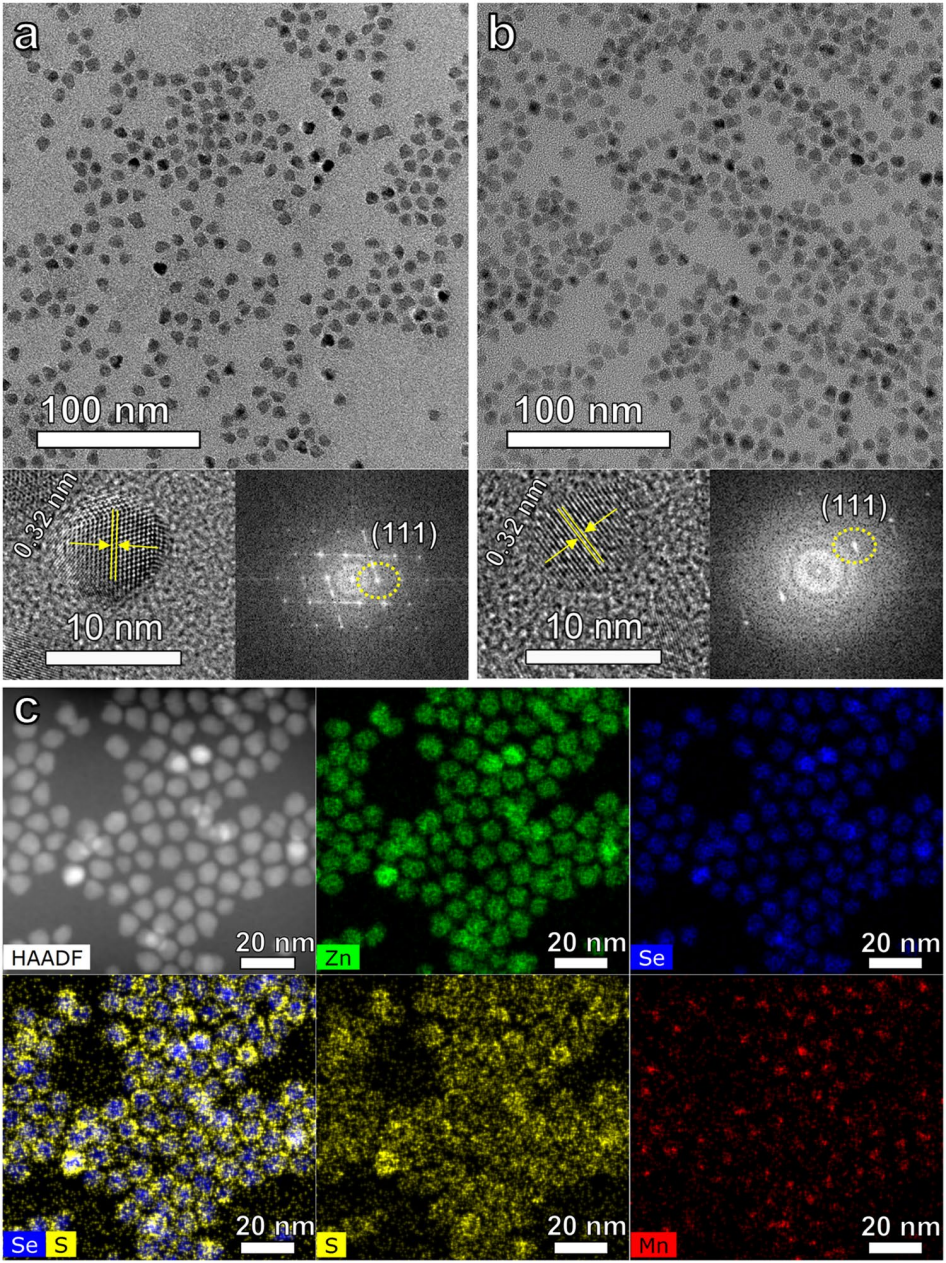
TEM images of (**a**) ZnSe:Mn^2+^(5%) d-C QDs and (**b**) ZnSe:Mn^2+^(5%)/ZnS d-C/S QDs. The left insets show HR-TEM images of (**a**) ZnSe:Mn^2+^(5%) d-C QDs and (**b**) ZnSe:Mn^2+^(5%)/ZnS d-C/S QDs, and the right insets show FFT patterns for the corresponding HR-TEM images. (**c**) HAADF-STEM image of the ZnSe:Mn^2+^(5%)/ZnS d-C/S QDs and EDS maps of Zn Kα (green), Se Kα (blue), S Kα (yellow), Mn Kα (red), and composite map of Se + S (blue + yellow).

In addition to the spectroscopic studies, X-ray diffraction (XRD) and transmission electron microscopy (TEM) were conducted to investigate the structure of the ZnSe:Mn^2+^(5%) d-C QDs and ZnSe:Mn^2+^(5%)/ZnS d-C/S QDs. Figure 4a and b exhibit TEM images of the ZnSe:Mn^2+^(5%) d-C QDs and ZnSe:Mn^2+^(5%)/ZnS d-C/S QDs. The size of the ZnSe:Mn^2+^(5%) d-C QDs was measured to be 7.1 ± 0.4 nm (average size ± standard deviation). The size was obtained by measuring the sizes of one hundred of ZnSe:Mn^2+^(5%) d-C QD particles shown in the TEM image. The ZnSe:Mn^2+^(5%)/ZnS d-C/S QDs showed an increased size of 7.8 ± 0.6 nm. Thus, the thickness of the ZnS shell on the d-C QD was calculated to be about 0.35 nm, indicating the formation of about 1 monolayer of the ZnS shell^42^. The high-resolution TEM (HR-TEM) images of the d-C QDs and d-C/S QDs exhibit clear lattice fringes, indicating the high crystallinity of the synthesized QDs. The fast Fourier transform (FFT) patterns of the synthesized QD samples showed spotty patterns, indicating the synthesis of single crystalline nanoparticles. These results account for the synthesis of single crystalline d-C and d-C/S QDs with high crystallinity. Using the FFT patterns, lattice spacings can be calculated and the lattice fringes of the d-C and d-C/S QDs were separated by 0.32 nm, which is consistent with interplanar spacing between the (111) planes of the zinc blende ZnSe crystal^27^. The formation of the cubic zinc blende structure was also confirmed by the XRD pattern of the ZnSe:Mn^2+^ d-C QDs. As shown in Fig. S2, three obvious XRD peaks can be observed at 27.2°, 45.5°, and 53.9°, and they correspond to the diffraction peaks for the (111), (220), and (311) planes of zinc blende ZnSe, respectively^27^. After the shelling procedure, the zinc blende structure was retained, and XRD peaks were slightly shifted to higher angles (27.5°, 45.7°, and 54°) due to the formation of the thin ZnS shell which has smaller lattice parameters than the ZnSe, and this result is consistent with the previous result reported by Zhu’s group^42^.

The growth of the ZnS shell on the ZnSe:Mn^2+^ d-C QD could be directly visualized by energy-dispersive X-ray spectroscopy (EDS) analysis. Figure 4c exhibits the high-angle annular dark-field (HAADF) scanning transmission electron microscopy (STEM) image, and the EDS maps which were obtained for the ZnSe:Mn^2+^/ZnS d-C/S QDs shown in the HAADF-STEM image. The EDS maps from Se and Mn clearly show that Se and Mn elements are confined to the core region, while those from Zn and S show that Zn and S occupied a wider region than Se and Mn. In particular, strong S signals were observed in the outer region of the QD particles in the S Kα map (Fig. 4c). The composite EDS map of the ZnSe:Mn^2+^/ZnS d-C/S QDs was produced by superposing the Se Kα and S Kα maps and it apparently shows the synthesis of core/shell-structured QDs. Consequently, the EDS results indicate that Mn^2+^ ions were doped into the ZnSe core and ZnSe:Mn^2+^/ZnS core/shell structure was successfully formed.

**Figure 5:**
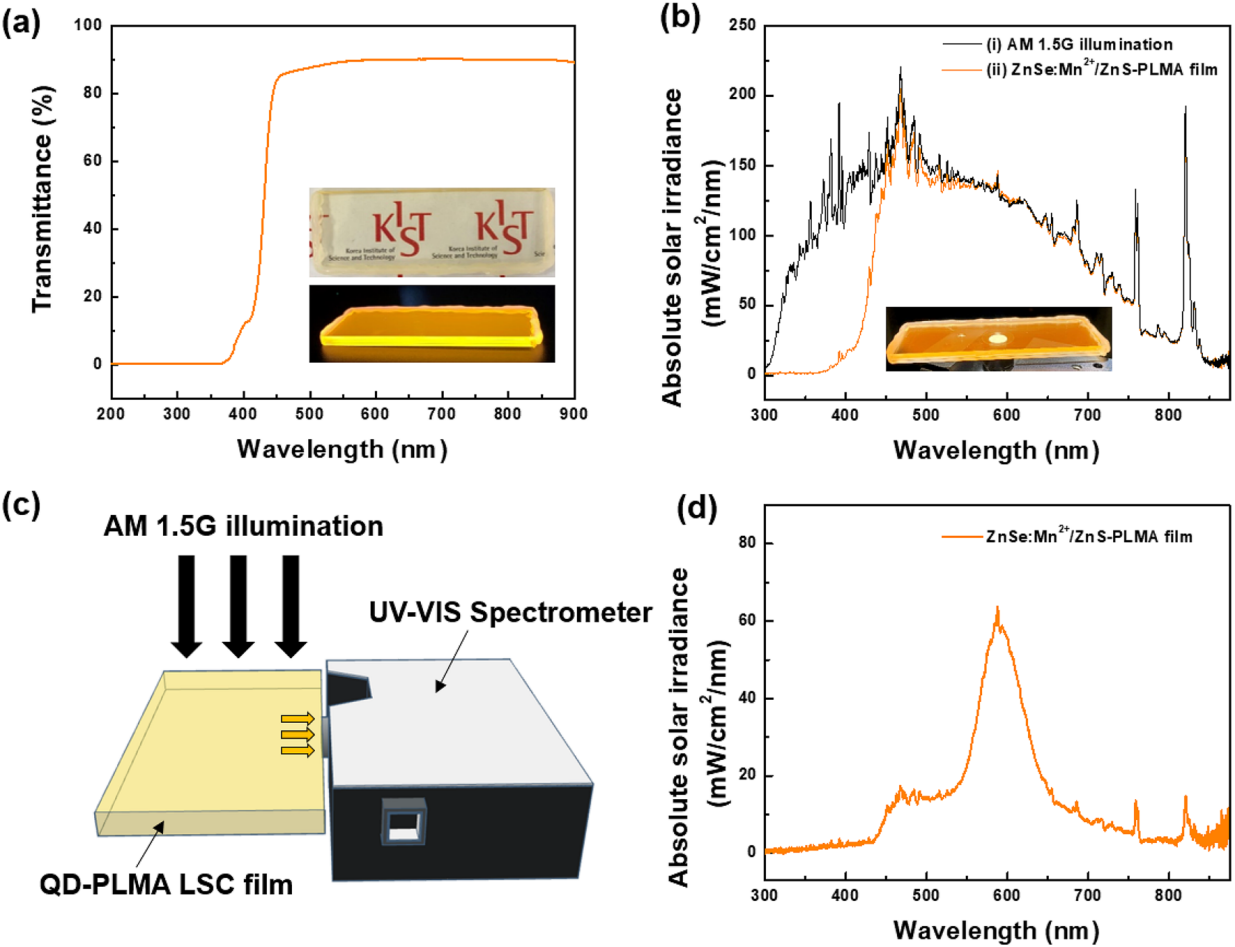
(**a**) Transmittance spectrum of ZnSe:Mn^2+^/ZnS-PLMA film. Inset shows photographs of the fabricated ZnSe:Mn^2+^/ZnS-PLMA film under indoor room light (top) and UV light (λ_ex_ = 365 nm) (bottom). (**b**) Irradiance spectra of (i) AM 1.5G light from a solar simulator and (ii) AM 1.5G light passing through the ZnSe:Mn^2+^/ZnS-PLMA film under AM 1.5G illumination from a solar simulator. Inset shows a photograph of the fabricated ZnSe:Mn^2+^/ZnS-PLMA film under AM 1.5G illumination. (**c**) Schematic illustration showing the measurement of the edge emission from the ZnSe:Mn^2+^/ZnS-PLMA LSC film. (d) Luminescence spectrum of the emission from the long edge of the ZnSe:Mn^2+^/ZnS-PLMA film under AM 1.5G illumination.

It is believed that the high PL QY of 83.3% and large Stokes shift of 170 nm of the ZnSe:Mn^2+^(5%)/ZnS d-C/S QDs are suitable for LSC applications. To assess the potential of the ZnSe:Mn^2+^/ZnS d-C/S QDs as luminophores for LSCs, a LSC film with dimensions of 76 mm × 26 mm × 2 mm (length × width × thickness) was fabricated by incorporating the ZnSe:Mn^2+^(5%)/ZnS d-C/S QDs into the PLMA polymer (Fig. S3). The as-fabricated ZnSe:Mn^2+^/ZnS-PLMA film exhibited high optical transparency, and the transmittance of the ZnSe:Mn^2+^/ZnS-PLMA film was higher than 83.8% for the visible spectral region longer than 450 nm (Fig. 5a). The transparent ZnSe:Mn^2+^/ZnS-PLMA film showed bright orange emission under a UV lamp (λ = 365 nm), as shown in Fig. 5a inset. Like the ZnSe:Mn^2+^/ZnS d-C/S QD solution, the ZnSe:Mn^2+^/ZnS-PLMA film showed a broad emission band peaking at 596 nm (Fig. S4). Importantly, the orange emission was clearly observed from the ZnSe:Mn^2+^/ZnS-PLMA film under outdoor natural sun light (Fig. S5).

Figure 5b shows the irradiance spectra from the air mass (AM) 1.5G solar simulator after passing through air and the ZnSe:Mn^2+^/ZnS-PLMA LSC film, respectively. The intensity of the solar irradiance dropped significantly below about 450 nm, which is similar to the decrease in transmittance in the similar wavelength range. This large decrease in solar irradiance is attributed to light absorption by the ZnSe:Mn^2+^/ZnS-PLMA film. As shown in Fig. 5b inset, the ZnSe:Mn^2+^/ZnS-PLMA film emitted orange light under AM 1.5G illumination from the solar simulator. However, the intensity of the ZnSe:Mn^2+^/ZnS-PLMA film at around 596 nm was similar to that of the AM 1.5G spectrum from the solar simulator, and this indicates that most of the light emitted from the ZnSe:Mn^2+^/ZnS QDs was guided to the edge of the film. As shown in Fig. 5c, when the emission from the ZnSe:Mn^2+^/ZnS-PLMA film was detected at the edge of the film, the apparent emission band was observed at around 596 nm under AM 1.5G illumination (Fig. 5d). When we compared emissions from long and short edges of the ZnSe:Mn^2+^/ZnS-PLMA film, the emission from the short edge of the ZnSe:Mn^2+^/ZnS-PLMA film showed higher intensity than that from the long edge of the ZnSe:Mn^2+^/ZnS-PLMA film (Fig. S6). The higher emission intensity can be attributed to large photon density at the short edge of the film due to smaller area of the short edge than that of the long edge of the film. However, if there is light loss owing to reabsorption of light emitted from the QDs in the LSC film, the emission from the short edge of the LSC film would not be stronger than that from the long edge of the LSC film. Thus, this result indicates that the light emitted from the ZnSe:Mn^2+^/ZnS-PLMA film exhibits minimal optical loss while propagating to the edge of the LSC film. The weak emission shoulder observed in the range from ~ 450 to 500 nm is attributed to the scattered light which was guided to the edge of the ZnSe:Mn^2+^/ZnS-PLMA film. The weak emission band due to the scattered light was also observed at the edge of the bare PLMA film without QDs when the bare PLMA film without QDs was illuminated with AM 1.5G white light from the solar simulator (Fig. S7).

**Figure 6:**
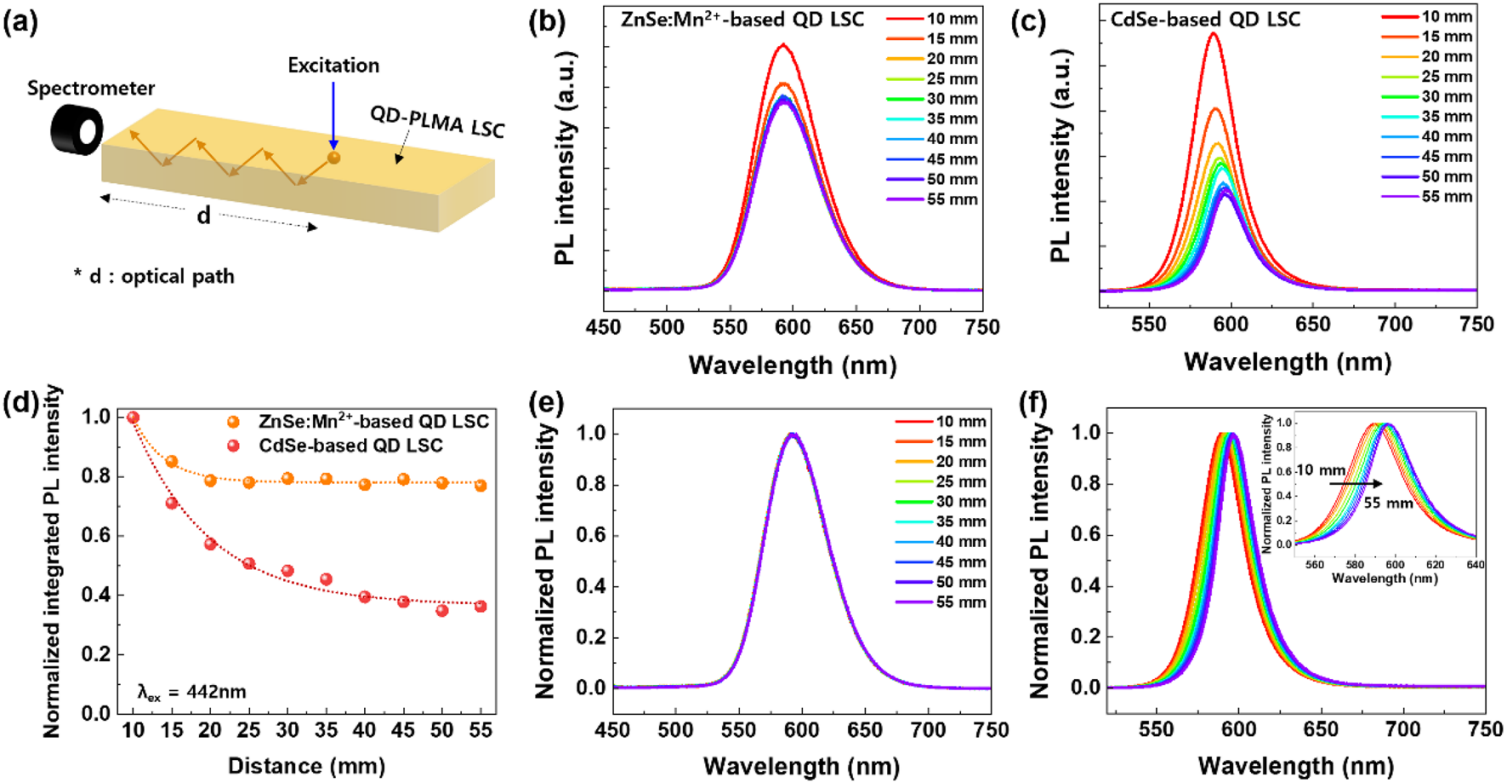
(**a**) Schematic illustration showing measurement of PL spectra from the QD-based LSC films while varying the position of excitation light, and the PL spectra of (**b**) the ZnSe:Mn^2+^/ZnS-PLMA LSC film and (**c**) CdSe/ZnS-PLMA LSC film while varying the distance of the excitation spot from the LSC edge. For optical path-dependent PL measurement, 442 nm light was used as an excitation source. (**d**) Normalized integrated PL intensity of the ZnSe:Mn^2+^/ZnS-PLMA LSC film (orange sphere) and the CdSe/ZnS-PLMA LSC film (red sphere), respectively, while varying the distance between the LSC edge and excitation spot. Normalized PL spectra of (**e**) the ZnSe:Mn^2+^/ZnS-PLMA LSC film and (f) the CdSe/ZnS-PLMA LSC film while varying the distance of an excitation spot from the LSC edge. Inset in (f) shows normalized PL spectra of the CdSe/ZnS-PLMA LSC film in the range from 550 to 640 nm.

To investigate the self-absorption loss of the ZnSe:Mn^2+^/ZnS-PLMA LSC film, optical path dependent edge PL spectra were obtained, as depicted in Fig. 6a. When the 442 nm light was used as an excitation source, apparent PL bands due to the ^4^T_1_ → ^6^A_1_ transition of Mn^2+^ ions were observed (Fig. 6b). Although PL intensity decreased with increasing distance between the edge of the LSC film and the position of the excitation light, it was saturated to 77.0% of the initial PL intensity and no further PL decrease was observed. For a control experiment, a CdSe/ZnS QD-PLMA LSC film was fabricated using commercial CdSe/ZnS QDs (Lumidot™, purchased from Sigma-Aldrich) since CdSe/ZnS QDs are representative QDs with high efficiency but small Stokes shift (Figs. S8 and S9). As shown in Fig. 6c, the PL intensity of the CdSe/ZnS-PLMA LSC film largely decreased when the distance between the edge of the LSC film and the position of the excitation light was increased. Notably, the PL intensity of the CdSe/ZnS-PLMA film rapidly decays due to the significant self-absorption loss, decreasing to about 36.3% of the initial PL intensity at d = 55 mm. Figure 6d exhibits normalized integrated PL intensities of the ZnSe:Mn^2+^/ZnS-PLMA and CdSe/ZnS-PLMA LSC films, and it shows that the optical path-dependent PL loss of the CdSe/ZnS-PLMA LSC film was much larger than that of the ZnSe:Mn^2+^/ZnS-PLMA LSC film. Importantly, no shift in PL band was observed for the ZnSe:Mn^2+^/ZnS-PLMA LSC film, regardless of the excitation position (Fig. 6e), while the CdSe/ZnS-PLMA LSC film showed significant redshift of the PL band depending on the excitation position (Fig. 6f). These results prove that there was no reabsorption in the ZnSe:Mn^2+^/ZnS-PLMA LSC film due to a large Stokes shift in the ZnSe:Mn^2+^/ZnS QDs. In contrast, while the light emitted from the CdSe/ZnS QDs propagated in the LSC film, the PL band largely shifted to a longer wavelength due to self-reabsorption by CdSe/ZnS QDs. Consequently, the optical path-dependent PL results of the QD-PLMA films confirm the advantage of ZnSe:Mn^2+^/ZnS QDs with large Stokes shift for LSC applications.

## Conclusion

In summary, cadmium-free and highly efficient orange-emitting ZnSe:Mn^2+^/ZnS d-C/S QDs were synthesized. The Mn^2+^ ion-doped ZnSe core was first synthesized via the nucleation doping method and the ZnS shell was grown on the ZnSe:Mn^2+^ core to reduce surface defects and increase exciton confinement. The generation of orange emission under excitation with UV light is attributed to the ^4^T_1_ → ^6^A_1_ transition of Mn^2+^ ions, and indicates Mn^2+^ was successfully doped into the ZnSe host. Also, EDS analysis directly showed Mn^2+^ doping into the ZnSe core and the successful formation of a core/shell structure. After the formation of the ZnS shell on the ZnSe:Mn^2+^ core, the PL QY of the ZnSe:Mn^2+^ significantly increased from 30.5 to 83.3%. The efficient ZnSe:Mn^2+^/ZnS d-C/S QDs exhibited a large Stokes shift of 170 nm which efficiently prevents the reabsorption of emission light from the ZnSe:Mn^2+^/ZnS d-C/S QDs. The ZnSe:Mn^2+^/ZnS-based LSC films were then prepared by incorporating the ZnSe:Mn^2+^/ZnS d-C/S QDs into PLMA polymer. The fabricated ZnSe:Mn^2+^/ZnS-PLMA LSC film showed high transparency in the visible spectral region and emitted bright orange light under AM 1.5G illumination using a solar simulator. The optical path-dependent PL properties of the QD-PLMA LSC film showed that the PL intensity of the ZnSe:Mn^2+^/ZnS-PLMA film slightly decreased and saturated at 77.0% of the initial PL intensity. Moreover, the ZnSe:Mn^2+^/ZnS-PLMA LSC film did not show any shift in PL band when the excitation position of the LSC film was varied. Consequently, the bright and large Stokes shift-emitting ZnSe:Mn^2+^/ZnS d-C/S QDs appear to be promising for application to LSCs.

## Methods

### Materials

Zinc oxide (ZnO, 99.99%), selenium (Se, 99.99%), sulfur (S, 99.9%), sodium oleate (C_18_H_33_NaO_2_, > 97.0%), manganese (II) chloride tetrahydrate (MnCl_2_·4H_2_O, ≥ 98%), trioctylphosphine (TOP, 90%), 1-octadecene (ODE, 90%), oleic acid (OA, 90%), hexane (95%), chloroform (95%), paraffin oil (PO, analytical grade), acetone (≥ 99.5%), methanol (MeOH, ≥ 99.8%), ethanol (EtOH, 99.5%), lauryl methacrylate (LMA, 96%), ethylene glycol dimethacrylate (EGDMA, 98%) and 2,2-Dimethoxy-2-phenylacetophenone (Irgacure 651, 99%) were purchased from Sigma-Aldrich.

### Preparation of the Zn precursor

A mixture of ZnO (0.1628 g, 2 mmol), OA (4.5 g, 16 mmol), and PO (14 ml) was loaded in a 50 mL three-neck flask and heated to 300 ℃ for 30 min under argon flow to obtain a colorless clear solution. After the reaction was completed, the temperature was cooled down to room temperature and the resulting Zn-oleate precursors were stored under argon atmosphere.

### Preparation of the Se precursor

A mixture of Se (0.1579 g, 2 mmol) and ODE (20 mL) was loaded in a 50 mL three-neck flask and heated to 220 ℃ for 180 min under argon flow. After the reaction was completed, the temperature was cooled down to room temperature and the Se precursors were stored under argon atmosphere.

### Preparation of the S precursor

A mixture of S (0.0644 g, 2 mmol) and TOP (20 mL) was loaded in a 50 mL three-neck flask and heated to 150 ℃ for 30 min under argon flow. After the reaction was completed, the temperature was cooled down to room temperature and the S precursors were stored under argon atmosphere.

### Preparation of the Mn precursor

The Mn precursor was prepared by modifying a previous method for the preparation of manganese stearate^25^. A mixture of sodium oleate (2.5 g, 8.212 mmol) and MeOH (40 mL) was loaded in a 100 mL three-neck flask and heated to 50 ℃ for 30 min. After the reaction was completed, MnCl_2_·4H_2_O (0.823 g, 4.158 mmol) in MeOH (5 ml) was injected dropwise. The temperature was cooled down to room temperature and the manganese oleate (Mn(OL)_2_) was washed with hexane and MeOH. The prepared Mn(OL)_2_ [C_36_H_66_MnO_4_ = 617.84 g/mol] (0.409 g, 0.662 mmol) and PO (26 mL) were loaded in a 50 mL three-neck flask followed by heat-treatment at 150 °C for 30 min under argon atmosphere, and the solution was stored under argon atmosphere.

### Synthesis of the ZnSe:Mn^2+^ d-C QDs

The ZnSe:Mn^2+^ d-C QDs were synthesized via the nucleation-doping process reported by Peng’s group with slight modification^25^. The Se precursor (2 mL), PO (4.5 mL), and Mn precursor, whose amount was dependent on doping concentration, were loaded in a 50 mL three-neck flask and heated to 150 ℃ for 30 min under vacuum. After degassing, the reaction temperature was increased to 260 ℃ under argon gas flow for 1 h. Then, the temperature of the reaction solution was increased to 310 ℃. As the reaction temperature increased, the Zn precursor solution (3 mL) was swiftly injected at 280 °C and then the temperature was kept at 310 ℃ for 1 h. Aliquots were taken at different time intervals to investigate the growth kinetics via optical property characterization such as UV–vis absorption spectroscopy and PL spectroscopy.

### Synthesis of the ZnSe:Mn^2+^/ZnS d-C/S QDs

One mL S precursor was slowly injected dropwise into the core solution at 310 ℃. After 10 min, 1 mL Zn precursor was injected dropwise into the core solution. After aging the solution for 60 min under argon atmosphere, the synthesized ZnSe:Mn^2+^/ZnS d-C/S QDs were washed by acetone, acetone/EtOH, and EtOH, and finally dispersed in hexane for further study.

### Fabrication of the ZnSe:Mn^2+^/ZnS d-C/S QD-based LSC film

The ZnSe:Mn^2+^(5%)/ZnS QD-incorporated LSC film was fabricated using LMA, EGDMA, and TOP, as reported by Patrick’s group^22^. First, the LMA mixture was prepared by mixing LMA, TOP, and EGDMA at a weight ratio of 5:1:1 and it was mixed with 1 wt.% of Irgacure 651 photoinitiator. After that, the ZnSe:Mn^2+^(5%)/ZnS QDs dispersed in hexane were dispersed in the LMA mixture. The hexane was removed via vacuum treatment and 1.3 wt.% ZnSe:Mn^2+^(5%)/ZnS-LMA mixture was prepared. The ZnSe:Mn^2+^(5%)/ZnS-PLMA film was fabricated by placing the ZnSe:Mn^2+^(5%)/ZnS-LMA mixture in the rectangular-shaped mold prepared with microscope slide glasses (76 mm × 26 mm × 2 mm) followed by curing with 306 nm UV light for 1 h.

### Characterization

The absorption and PL spectra of the ZnSe:Mn^2+^-based QDs were collected with a PerkinElmer lambda25 spectrometer and a Hitachi F-7000 spectrophotometer, respectively. The size and morphologies of the QDs were investigated by TEM using an FEI Tecnai F20 G^2^ transmission electron microscope operated at 200 kV. The EDS mapping was conducted using an FEI Talos 200X transmission electron microscope operated at 200 kV. The crystal structure of the QDs was analyzed by XRD using a Bruker D8-Advance operated at 40 kV and 40 mA (λ_CuKα_ = 1.5406 Å). The absolute PL QYs of the ZnSe:Mn^2+^ d-C QDs and ZnSe:Mn^2+^/ZnS d-C/S QDs were measured with a QE-1100 quantum efficiency measurement system using an integrating hemisphere (Otsuka Electronics Co. Ltd., Japan). The luminescence properties of the ZnSe:Mn^2+^/ZnS-PLMA film were characterized with a spectrometer (USB4000, Ocean Optics, Inc., USA) under white light illumination using an AM 1.5G solar simulator (SAN-EI electric co., Ltd, Osaka, Japan).

## Supplementary Information

Supplementary Information.

## Data Availability

The datasets used and/or analyzed during the current study available from the corresponding author on reasonable request.

## References

[1] Debije MG, Verbunt PPC (2012). Thirty years of luminescent solar concentrator research: solar energy for the built environment. Adv. Energy Mater..

[2] Swartz BA, Cole T, Zewail AH (1977). Photon trapping and energy transfer in multiple-dye plastic matrices: an efficient solar-energy concentrator. Opt. Lett..

[3] Debije MG (2011). Promising fluorescent dye for solar energy conversion based on a perylene perinone. Appl. Opt..

[4] Li C (2015). Large stokes shift and high efficiency luminescent solar concentrator incorporated with CuInS_2_/ZnS quantum dots. Sci. Rep..

[5] Zhao H (2016). Solar concentrators: absorption enhancement in “giant” core/alloyed-shell quantum dots for luminescent solar concentrator. Small.

[6] Bomm J (2011). Fabrication and full characterization of state-of-the-art quantum dot luminescent solar concentrators. Sol. Energy Mater. Sol. Cells.

[7] Zhao H, Zhou Y, Benetti D, Ma D, Rosei F (2017). Perovskite quantum dots integrated in large-area luminescent solar concentrators. Nano Energy.

[8] Coropceanu I, Bawendi MG (2014). Core/shell quantum dot based luminescent solar concentrators with reduced reabsorption and enhanced efficiency. Nano Lett..

[9] Meinardi F (2014). Large-area luminescent solar concentrators based on ‘Stokes-shift-engineered’ nanocrystals in a mass-polymerized PMMA matrix. Nat. Photonics.

[10] Bronstein ND (2014). Luminescent solar concentration with semiconductor nanorods and transfer-printed micro-silicon solar cells. ACS Nano.

[11] Li H, Wu K, Lim J, Song H-J, Klimov VI (2016). Doctor-blade deposition of quantum dots onto standard window glass for low-loss large-area luminescent solar concentrators. Nat. Energy.

[12] Meinardi F (2015). Highly efficient large-area colourless luminescent solar concentrators using heavy-metal-free colloidal quantum dots. Nat. Nanotechnol..

[13] Sumner R (2017). Analysis of optical losses in high-efficiency CuInS_2_-based nanocrystal luminescent solar concentrators: balancing absorption versus scattering. J. Phys. Chem. C.

[14] Meinardi F (2017). Highly efficient luminescent solar concentrators based on earth-abundant indirect-bandgap silicon quantum dots. Nat. Photonics.

[15] Pradhan N, Goorskey D, Thessing J, Peng X (2005). An Alternative of CdSe nanocrystal emitters: pure and tunable impurity emissions in ZnSe nanocrystals. J. Am. Chem. Soc..

[16] Thuy UTD, Maurice A, Liem NQ, Reiss P (2013). Europium doped In(Zn)P/ZnS colloidal quantum dots. Dalton Trans..

[17] Vlaskin VA, Janssen N, van Rijssel J, Beaulac R, Gamelin DR (2010). Tunable dual emission in doped semiconductor nanocrystals. Nano Lett..

[18] Pan G (2017). Doping lanthanide into perovskite nanocrystals: highly improved and expanded optical properties. Nano Lett..

[19] Wu K, Li H, Klimov VI (2018). Tandem luminescent solar concentrators based on engineered quantum dots. Nat. Photonics.

[20] Bradshaw LR, Knowles KE, McDowall S, Gamelin DR (2015). Nanocrystals for luminescent solar concentrators. Nano Lett..

[21] Luo X, Ding T, Liu X, Liu Y, Wu K (2019). Quantum-cutting luminescent solar concentrators using ytterbium-doped perovskite nanocrystals. Nano Lett..

[22] Erickson CS (2014). Zero-reabsorption doped-nanocrystal luminescent solar concentrators. ACS Nano.

[23] Reiss P (2007). ZnSe based colloidal nanocrystals: synthesis, shape control, core/shell, alloy and doped systems. New J. Chem..

[24] Gul S (2013). Effect of Al^3+^ co-doping on the dopant local structure, optical properties, and exciton dynamics in Cu^+^-doped ZnSe nanocrystals. ACS Nano.

[25] Pradhan N, Peng X (2007). Efficient and color-tunable Mn-doped ZnSe nanocrystal emitters: control of optical performance via greener synthetic chemistry. J. Am. Chem. Soc..

[26] Zhou R (2018). Enriching Mn-doped ZnSe quantum dots onto mesoporous silica nanoparticles for enhanced fluorescence/magnetic resonance imaging dual-modal bio-imaging. ACS Appl. Mater. Interfaces.

[27] Khan ZU (2020). Orange-emitting ZnSe:Mn^2+^ quantum dots as nanoprobes for macrophages. ACS Appl. Nano Mater..

[28] Byambasuren N (2021). Phosphine-free-synthesized ZnSe/ZnS Core/shell quantum dots for white light-emitting diodes. Appl. Sci..

[29] Li F, Xia Z, Liu Q (2017). Controllable synthesis and optical properties of ZnS:Mn^2+^/ZnS/ZnS:Cu^2+^/ZnS core/multishell quantum dots toward efficient white light emission. ACS Appl. Mater. Interfaces.

[30] Zheng J (2010). Improved photoluminescence of MnS/ZnS core/shell nanocrystals by controlling diffusion of Mn Ions into the ZnS shell. J. Phys. Chem. C.

[31] Zeng R, Zhang T, Dai G, Zou B (2011). Highly emissive, color-tunable, phosphine-free Mn:ZnSe/ZnS core/shell and Mn:ZnSeS shell-alloyed doped nanocrystals. J. Phys. Chem. C.

[32] Selvaraj J (2017). Phosphine-free, highly emissive, water-soluble Mn:ZnSe/ZnS core-shell nanorods: synthesis, characterization, and in vitro bioimaging of HEK293 and HeLa cells. ACS Appl. Nano Mater..

[33] Yang X (2019). Temperature- and Mn^2+^ concentration-dependent emission properties of Mn^2+^-doped ZnSe nanocrystals. J. Am. Chem. Soc..

[34] Jin X (2018). Stretchable silica gel-ZnSe:Mn/ZnS quantum dots for encoding. Opt. Mater. Express.

[35] Song S-W (2022). Single and dual doping of blue-emissive ZnSeTe quantum dots with transition metal ions. Adv. Opt. Mater..

[36] Cao S (2013). Highly efficient and well-resolved Mn^2+^ ion emission in MnS/ZnS/CdS quantum dots. J. Mater. Chem. C.

[37] Chauhan H, Kumar Y, Deka S (2014). New synthesis of two-dimensional CdSe/CdS core@shell dot-in-hexagonal platelet nanoheterostructures with interesting optical properties. Nanoscale.

[38] Aboulaich A (2010). Water-based route to colloidal Mn-doped ZnSe and core/shell ZnSe/ZnS quantum dots. Inorg. Chem..

[39] Sharma VK, Guzelturk B, Erdem T, Kelestemur Y, Demir HV (2014). Tunable white-light-emitting Mn-doped ZnSe nanocrystals. ACS Appl. Mater. Interfaces.

[40] Ishizumi A, Jojima E, Yamamoto A, Kanemitsu Y (2008). Photoluminescence dynamics of Mn^2+^-Doped CdS/ZnS core/shell nanocrystals: Mn^2+^ concentration dependence. J. Phys. Soc. Jpn..

[41] Gan C, Zhang Y, Battaglia D, Peng X, Xiao M (2008). Fluorescence lifetime of Mn-doped ZnSe quantum dots with size dependence. Appl. Phys. Lett..

[42] Fang Z, Li Y, Zhang H, Zhong X, Zhu L (2009). Facile synthesis of highly luminescent UV-blue-emitting ZnSe/ZnS core/shell nanocrystals in aqueous media. J. Phys. Chem. C.

