# Supplementary material for: Environmentally friendly, highly efficient, and large stokes shift-emitting ZnSe:Mn2+/ZnS core/shell quantum dots for luminescent solar concentrators
Source: Sci Rep. 2022 Oct 20;12:17595. doi: 10.1038/s41598-022-21090-x (PMC9584966; doi:10.1038/s41598-022-21090-x)
Supplement: Supplementary file 1 — Supplementary Information. [file 41598_2022_21090_MOESM1_ESM.pdf]

Supplementary Information

Environmentally friendly, highly efficient, and large Stokes shift-emitting ZnSe:Mn^{2+}/ZnS core/shell quantum dots for luminescent solar concentrators

Nyamsuren Byambasuren[1,2], A-Ra Hong[1], Woo-Young Lee[3], Ji Young Byun[4], Gumin Kang[3,*], Hyungduk Ko[3,*] & Ho Seong Jang[1,2,*]

[1]Materials Architecturing Research Center, Korea Institute of Science and Technology, 5, Hwarang-ro 14-gil, Seongbuk-gu, Seoul 02792, Republic of Korea

[2]Division of Nano & Information Technology, KIST School, Korea University of Science and Technology (UST), Seoul 02792, Republic of Korea

[3]Nanophotonics Research Center, Korea Institute of Science and Technology, 5, Hwarang-ro 14-gil, Seongbuk-gu, Seoul 02792, Republic of Korea

[4]Extreme Materials Research Center, Korea Institute of Science and Technology, 5, Hwarang-ro 14-gil, Seongbuk-gu, Seoul 02792, Republic of Korea

To whom correspondence should be addressed.

E-mail: msekorea@kist.re.kr (H. S. Jang), kohd94@kist.re.kr (H. Ko), guminkang@kist.re.kr (G. Kang)

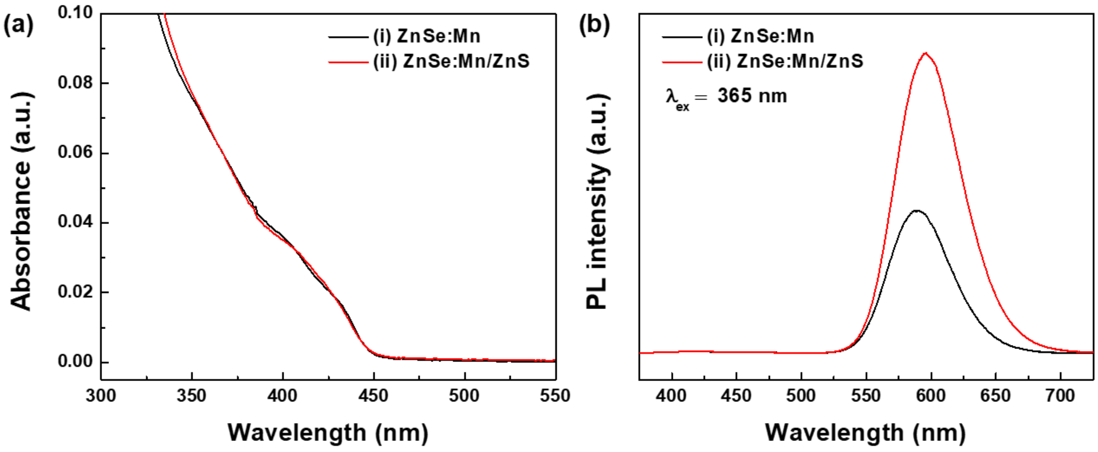

Figure S1. (a) Absorption and (b) PL spectra of (i) ZnSe:Mn^{2+}(5%) d-C QDs and (ii) ZnSe:Mn^{2+}(5%)/ZnS d-C/S QDs.

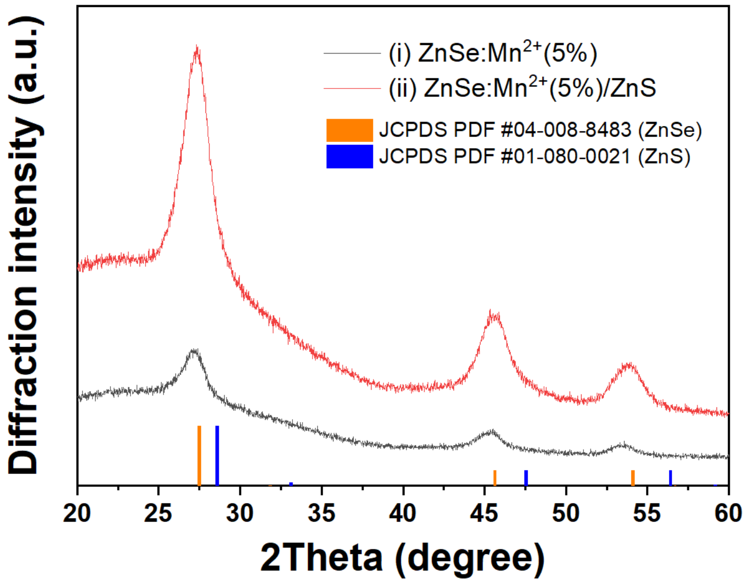

Figure S2. XRD Patterns of (i) ZnSe:Mn^{2+}(5%) d-C QDs (black line) and (ii) ZnSe:Mn^{2+}(5%)/ZnS d-C/S QDs (red line)

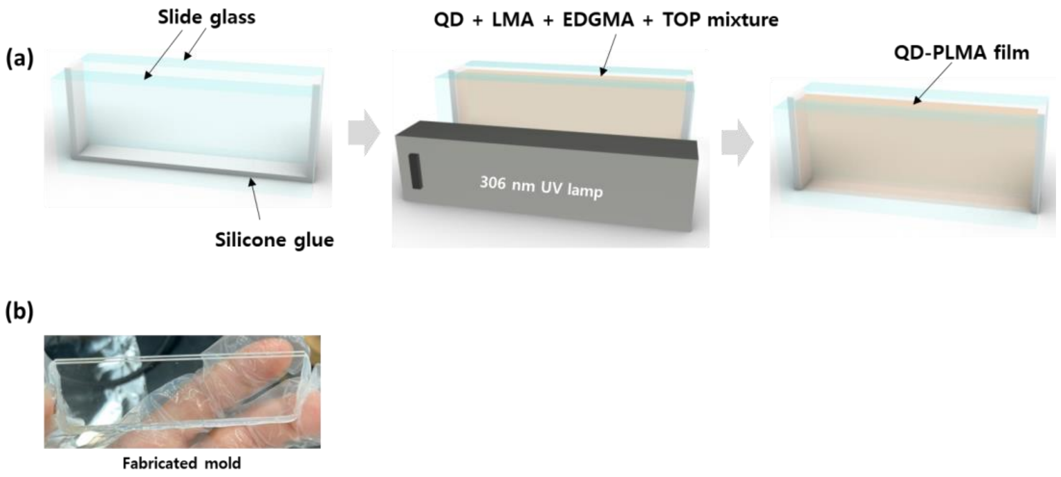

Figure S3. (a) Schematic illustration showing the fabrication of the ZnSe:Mn^{2+}/ZnS-PLMA film and (b) the photograph of the mold for LSC films fabricated with slide glasses.

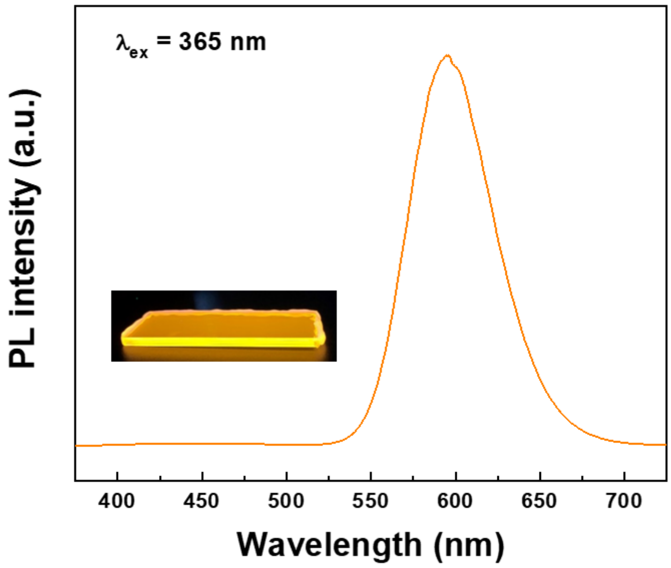

Figure S4. PL spectrum of the ZnSe:Mn^{2+}/ZnS-PLMA film under 365 nm UV light. Inset shows the photograph showing the luminescence from the ZnSe:Mn^{2+}/ZnS-PLMA film under a hand-held UV lamp (λ = 365 nm).

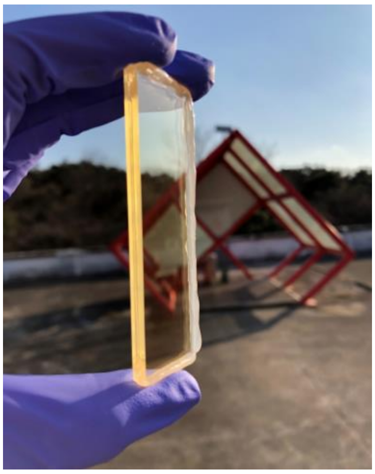

Figure S5. Photograph showing the luminescence from the ZnSe:Mn^{2+}/ZnS-PLMA film under outdoor natural sunlight.

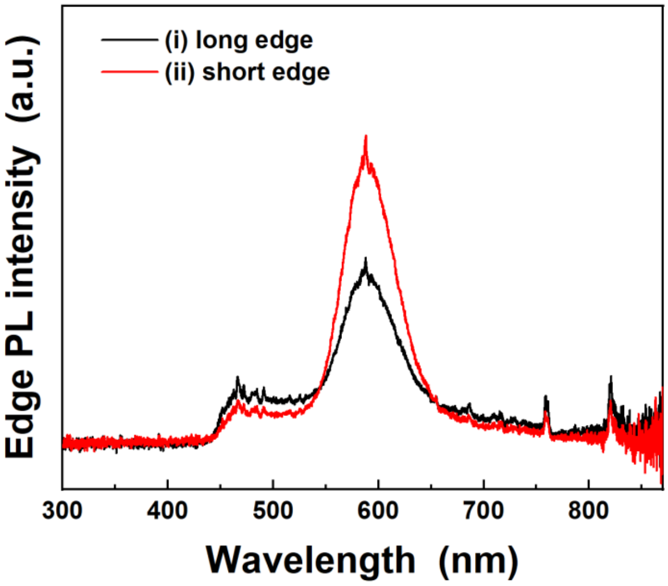

Figure S6. Luminescence spectra of the edge emissions from (i) long and (ii) short edges of the ZnSe:Mn^{2+}/ZnS-PLMA film under AM 1.5G illumination.

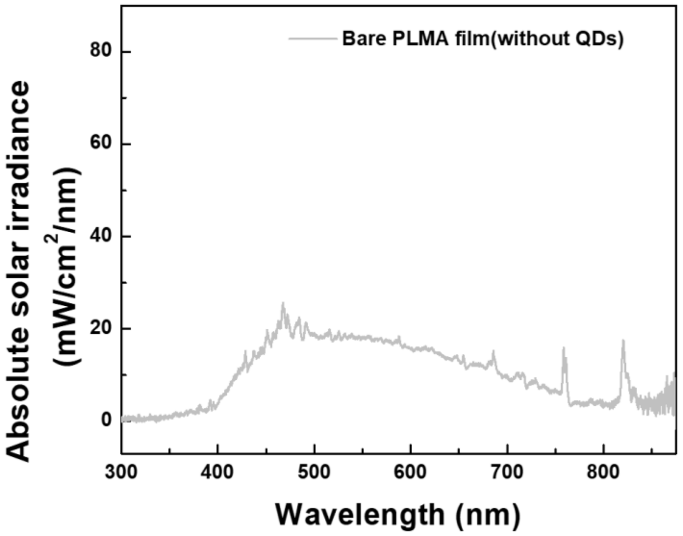

Figure S7. Luminescence spectrum of the bare PLMA film under AM 1.5G illumination.

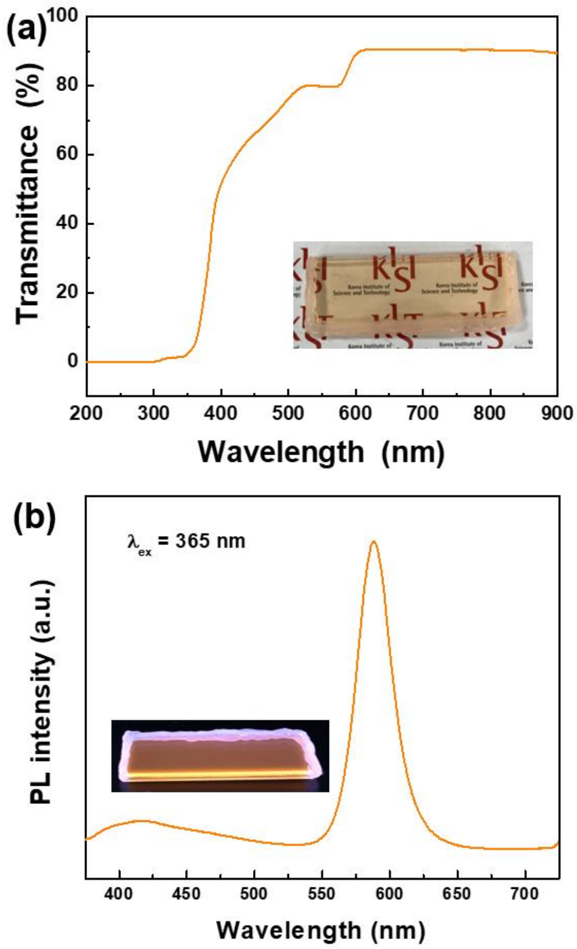

Figure S8. (a) Tansmittance spectrum of the CdSe/ZnS-PLMA film. Inset shows the photograph of the CdSe/ZnS-PLMA film under indoor room light. (b) PL spectrum of the CdSe/ZnS-PLMA film under 365 nm UV light. Inset shows the photograph showing the luminescence from the CdSe/ZnS-PLMA film under a hand-held UV lamp (λ = 365 nm).

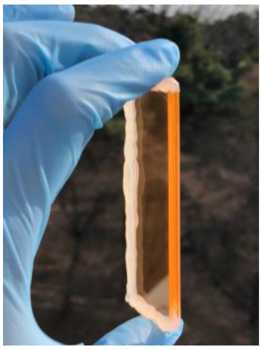

Figure S9. Photograph showing the luminescence from the CdSe/ZnS-PLMA film under outdoor natural sunlight.
